# Unsupervised machine learning analysis to enhance risk stratification in patients with asymptomatic aortic stenosis

**DOI:** 10.1093/ehjdh/ztaf115

**Published:** 2025-10-09

**Authors:** Marie-Ange Fleury, Louis Ohl, Lionel Tastet, Mickaël Leclercq, Frédéric Precioso, Pierre-Alexandre Mattei, Romain Capoulade, Kathia Abdoun, Élisabeth Bédard, Marie Arsenault, Jonathan Beaudoin, Mathieu Bernier, Erwan Salaun, Jérémy Bernard, Mylène Shen, Sébastien Hecht, Nancy Côté, Arnaud Droit, Philippe Pibarot

**Affiliations:** Institut universitaire de cardiologie et de pneumologie de Québec, Université Laval, Laval University, 2725 Chemin Sainte-Foy, Quebec city, Québec G1V-4G5, Canada; Department of Computer and Information Science, STIMA—Linköping University, Linköping, Sweden; Department of Medicine, Cardiovascular Division, University of California San Francisco, San Francisco, CA, USA; Computational biology, Faculté de médecine, Université Laval, CHU de Québec Research Center, 2705 boul. Laurier, Québec, QC G1V 4G2, Canada; Inria, Maasai team—Université Côte d’Azur, Nice, France; Inria, Maasai team—Université Côte d’Azur, Nice, France; Nantes Université, CHU Nantes, CNRS, INSERM, l’institut du thorax, Nantes F-44000, France; Institut universitaire de cardiologie et de pneumologie de Québec, Université Laval, Laval University, 2725 Chemin Sainte-Foy, Quebec city, Québec G1V-4G5, Canada; Institut universitaire de cardiologie et de pneumologie de Québec, Université Laval, Laval University, 2725 Chemin Sainte-Foy, Quebec city, Québec G1V-4G5, Canada; Institut universitaire de cardiologie et de pneumologie de Québec, Université Laval, Laval University, 2725 Chemin Sainte-Foy, Quebec city, Québec G1V-4G5, Canada; Institut universitaire de cardiologie et de pneumologie de Québec, Université Laval, Laval University, 2725 Chemin Sainte-Foy, Quebec city, Québec G1V-4G5, Canada; Institut universitaire de cardiologie et de pneumologie de Québec, Université Laval, Laval University, 2725 Chemin Sainte-Foy, Quebec city, Québec G1V-4G5, Canada; Institut universitaire de cardiologie et de pneumologie de Québec, Université Laval, Laval University, 2725 Chemin Sainte-Foy, Quebec city, Québec G1V-4G5, Canada; Institut universitaire de cardiologie et de pneumologie de Québec, Université Laval, Laval University, 2725 Chemin Sainte-Foy, Quebec city, Québec G1V-4G5, Canada; Institut universitaire de cardiologie et de pneumologie de Québec, Université Laval, Laval University, 2725 Chemin Sainte-Foy, Quebec city, Québec G1V-4G5, Canada; Institut universitaire de cardiologie et de pneumologie de Québec, Université Laval, Laval University, 2725 Chemin Sainte-Foy, Quebec city, Québec G1V-4G5, Canada; Institut universitaire de cardiologie et de pneumologie de Québec, Université Laval, Laval University, 2725 Chemin Sainte-Foy, Quebec city, Québec G1V-4G5, Canada; Computational biology, Faculté de médecine, Université Laval, CHU de Québec Research Center, 2705 boul. Laurier, Québec, QC G1V 4G2, Canada; Institut universitaire de cardiologie et de pneumologie de Québec, Université Laval, Laval University, 2725 Chemin Sainte-Foy, Quebec city, Québec G1V-4G5, Canada

**Keywords:** Unsupervised machine learning, Clustering, Aortic stenosis, Echocardiography, Risk stratification

## Abstract

**Aims:**

There is a lack of studies investigating the pathophysiologic and phenotypic distinctiveness of aortic stenosis (AS). This heterogeneity has important implications for identifying optimal intervention timing and potential medical management. This study seeks to identify phenogroups of AS using unsupervised machine learning to improve risk stratification.

**Methods and results:**

A total of 349 patients with asymptomatic AS from the PROGRESSA study were included in this analysis. Echocardiographic, clinical and blood sample data were used in the unsupervised clustering process. Longitudinal echocardiographic data were used to evaluate AS progression. Five clusters of patients were revealed using 18 variables selected by an unsupervised machine learning algorithm. Amongst them, aortic valvular phenotype, mean gradient, peak jet velocity (V_peak_), and left ventricle stroke volume were selected as discriminatory variables. Following the clustering process, characteristics differed between clusters, including age, body mass index, and sex ratio (all *P* < 0.001). Of note, cluster 1 showed higher AS severity at baseline with significantly higher initial V_peak_ (344 [314; 376] cm/s) and calcium score (1257 [806; 1837] UA) (*P* < 0.001). Patients from cluster 1 had a faster AS progression (progression of V_peak_ = 22 [9; 39] cm/s/year), and calcium score (213 [111; 307] UA/year) (*P* < 0.001). Cluster 1 was also associated with a higher composite risk of mortality and aortic valve replacement when adjusted for age, sex, and baseline AS severity (*P* < 0.001).

**Conclusion:**

Artificial intelligence-guided phenotypic classification revealed 5 distinct groups and enhanced risk stratification of patients with AS. This approach may be useful to optimize and individualize medical and interventional management of AS.

## Graphical Abstract

**Graphical Abstract:**
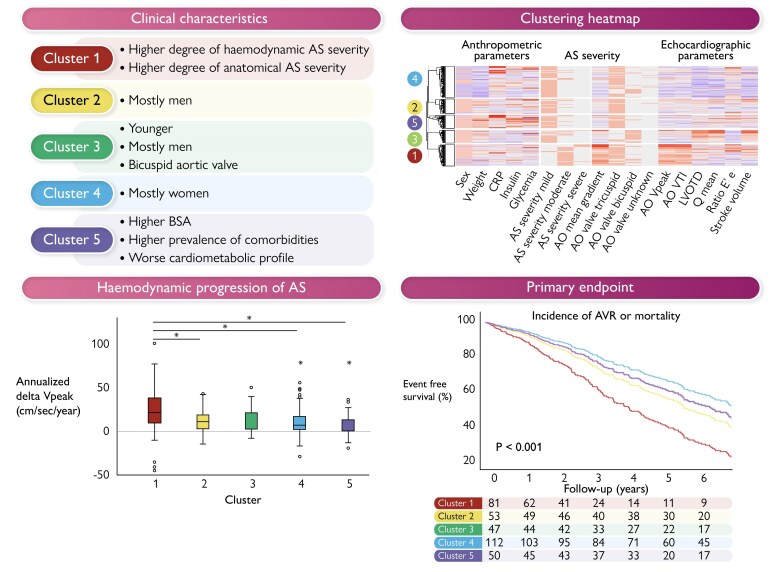

## Introduction

Aortic stenosis (AS) is the most prevalent valvular heart disease in high-income countries,^1–4^ and 3 million individuals are affected by AS in North America, increasing prevalence related to the population’s aging.^3,5^ To date, no effective pharmacological treatment to prevent the development and/or progression of AS exists. Aortic valve replacement (AVR) by surgery (SAVR) or transcatheter implantation (TAVI) remains the only therapeutic options for patients with symptomatic severe AS.^6,7^ Currently, AS is classified into four stages, primarily based on echocardiographic haemodynamic assessments of the aortic valve.^8^ However, the progression of AS is highly variable and influenced by patient-specific factors, including both cardiac and non-cardiac conditions.^9^ In patients with AS, current guidelines recommendations rely essentially on the assessment of AS severity, symptom status, and left ventricular (LV) systolic function (LVEF <50%) to indicate intervention. However, the literature documents that the presence of cardiac damage beyond the valve can vary substantially among patients and holds prognostic significance.^10–12^ Thus, the current ‘valve-centric’ classification may not fully integrate the complexity of AS and cardiac damage, and incorporate a more detailed, multi-factor phenotyping approach that could provide added value.

Several recent studies have reported the applicability and accuracy of machine and deep learning-based algorithms to detect AS in various settings.^13–16^ In addition, we previously demonstrated the usefulness of a novel machine-learning pipeline that integrates echocardiographic parameters to improve risk stratification of AS.^17^ Approaches for identifying phenogroups, i.e. the clustering task, have already been applied in AS.^17–20^ However, these studies often seek clusters that are only related to either the severity of the AS or the expected survival rate of patients and do not focus on the identification of groups of patients displaying different traits yet undergoing the same disease.

To optimize risk stratification and patient management, we hypothesize that different phenogroups exist amongst AS patients. These phenotypes could potentially be non-mutually exclusive and help in determining the specific physiopathologic pathways of AS that are more present in specific patients. In the present study, a full pipeline for clustering that remains stable over time was developed. The key hypothesis for this requirement is that phenogroups do not change over time thus patients belong only to a specific phenogroup. The aims of this study were: (i) to identify and describe phenotypes of AS and (ii) to assess phenotype-specific clinical outcomes.

## Methods

### Study population

A cohort of 349 patients with at least mild asymptomatic AS (i.e. Vpeak ≥ 200 cm/s) were prospectively recruited in the PROGRESSA (Metabolic Determinants of the Progression of AS) study (NCT01679431) between 2005 and 2020, at the Institut universitaire de cardiologie et de pneumologie de Québec—Université Laval (IUCPQ-ULaval) and underwent yearly follow-up visits. The purpose and design of the PROGRESSA study have been previously described.^21,22^ Patients were excluded if they had symptomatic AS, pre-existing AVR requirement, moderate or greater aortic regurgitation, mitral valve disease (stenosis or regurgitation), left ventricular ejection fraction (LVEF) < 50%, and if they were pregnant or lactating.

### Consent

The study was approved by the Ethics Committee of the IUCPQ-ULaval and all patients signed a written informed consent form at the time of enrolment.

### Clinical and laboratory data

Clinical data used in this analysis has been previously described.^23^ From fasting blood samples, plasma levels of glucose, creatinine, N-terminal pro B-type natriuretic peptide (Nt-pro-BNP), high-sensitivity troponin T, standard lipid profile, apolipoprotein B (apo B), apolipoprotein A-I (apo A-I), C-reactive protein (CRP), and standard haematology profile were measured using automated techniques standardized by the Canadian reference laboratory (see Supplementary material online, *Table S1*). CRP has been previously associated with AS and in this study, it was used as a marker for inflammation and sheer stress.^24^

### Echocardiographic data

Comprehensive Doppler echocardiography exams were performed using commercially available ultrasound systems. All echocardiographic exams were conducted by the same team of sonographers and cardiologists; all images were analyzed by experienced readers. Doppler echocardiography methods were described in a previous study.^21^ AS severity was classified as mild (V_peak_ 200–290 cm/s), moderate (V_peak_ 300–390 cm/s), or severe (V_peak_ ≥400 cm/s). AS progression was determined using subsequent echocardiographic examinations and annualized to accommodate for the various follow-up times of the patients included in this analysis (Last follow-up measurement—baseline measurement/Time between baseline and last follow-up) (see Supplementary material online, *Table S2*).

### Computed tomography

The protocol for multidetector CT (MDCT) image acquisition and interpretation was previously published.^25,26^ Aortic valve calcium (AVC) score data was obtained by using the Aquarius (iNtuition, TeraRecon, California, U.S.A.) software for analysis. All scores were quantified with the Agatston method and are expressed in the arbitrary unit (AU) (see Supplementary material online, *Table S2*).

### Study endpoints

The primary endpoints of the present study were: (ⅰ) all-cause mortality (*n* = 66, 19%) and (ⅱ) a competitive composite of all-cause mortality and AVR (*n* = 188, 54%) either by SAVR or TAVI.

### Clustering process

**Figure 1:**
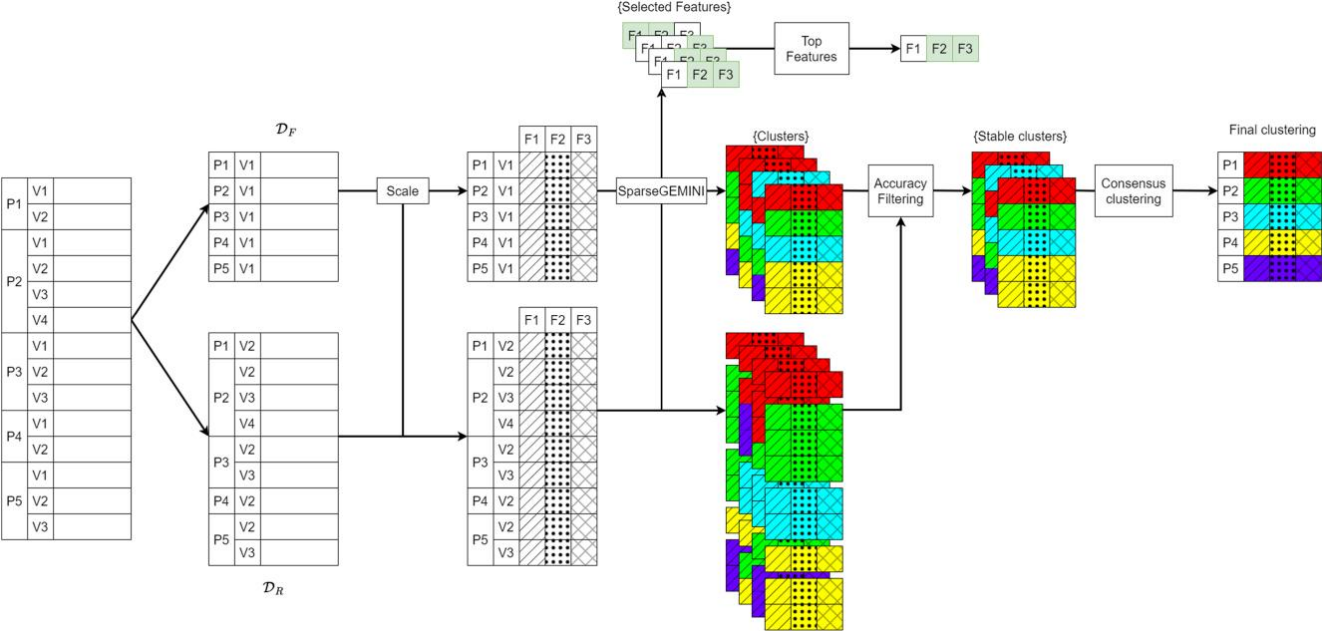
Complete clustering process of the PROGRESSA dataset with stable clusters over different patient visits with feature selection. The data is first split between the dataset of the first visit (Df) and the dataset of remaining visits (Dr). Then, after scaling, SparseGEMINI is applied multiple times to obtain a set of clusters for both datasets and a set of selected features. Selected features are used to evaluate the most frequently used features. Clusters of subsequent visits are used to filter models that always put subsequent visits of patients in the same cluster as the first visit. Consensus clustering is applied to the set of filtered and stable clusters to get a final clustering. Legend: P: patient; V: visit; F: feature, GEMINI: Generalized mutual information. For example, P2 is the second patient of the database, V3 is the third visit of a patient and F1 is the first feature of the dataset. Dashed areas correspond to scaled features. Colours correspond to an arbitrary cluster number, irrespective of the actual clustering results. Selected features by each model are coloured in green.

The complete clustering method is detailed in the Supplementary material. Briefly, the PROGRESSA study database was divided into two subsets: the subset of the first patients’ visit and the subset of the subsequent visits. To perform clustering, we sought to use a discriminative method and incorporated a feature selection mechanism inside the model to keep a few variables that are relevant for interpreting the clusters. The outcomes of patients was blinded during this process. We used the generalized mutual information^27^ (GEMINI) for clustering: an information-theoretic score for discriminative clustering. To conclude on a final clustering, consensus clustering was used.^28^ To identify stable-over-time clusters, each model was used to cluster the remaining visits of every patient. Then, the first visit cluster was used as a ground truth label, allowing us to measure the accuracy of the remaining visits’ clusters. Both the visit-wise accuracy and the patient-wise accuracy that accounts for the imbalance of visits between all patients are reported. Models that achieve a patient-wise accuracy within 90% of the best accuracy of clusters were kept. The complete picture of the clustering process is summarized in *Figure 1*. Since consensus clustering cannot generalize to unseen patients, we trained a logistic regression to map patients to the clusters. It is available upon reasonable request.

### Statistical analysis

Continuous variables were tested for normality by the Shapiro-Wilk or the Kolmogorov-Smirnov tests and presented as mean ± standard deviation or as median [interquartile range] if not normally distributed. Independent sample Kruskal Wallis tests were performed to evaluate differences between clusters. Categorical variables were expressed as number of patients and percent and compared using the chi-squared test. The association between clusters and all-cause mortality and the composite endpoint of all-cause mortality and AVR were assessed using Cox proportional hazards regression analyses. The estimates of cumulative incidence of events were calculated using the Kaplan-Meier method/curves and compared using the Log-rank test. The proportional hazards assumption was confirmed through the evaluation of scaled Schoenfeld residuals. Multivariate analyses were performed and adjusted for age, sex and initial V_peak_ to limit the influence of confounding factors. Statistical significance was defined as *P* < 0.05. Statistical analyses were performed using SPSS version 29.0 (IBM Corporation, Armonk, New York) and STATA version 17.0 (StataCorp, College Station, Texas).

## Results

### Study sample characteristics

The overall cohort characteristics can be found in Supplementary material online, *Table S1*. The median age was 68 [57–74] years, with 70% of male patients and a mean body mass index of 28.4 [25.6; 30.9]. Echocardiographic and MDCT data are presented in Supplementary material online, *Table S2*. Most patients had mild AS (67%) and mean V_peak_ and AVC score were 269 [242–309] and 600 [295–1075], respectively.

### Pipeline metrics

The preprocessing of the dataset returned 349 patients, i.e. 349 first visits, with 87 variables converted to 95 features. The set of subsequent visits contained 1196 samples. After executing the pipeline, the 120 models were filtered to a subset of 33 more accurate models. Their visit-wise accuracy is 67.5% [CI95%: 66.7%–68.3%] and their patient-wise accuracy is 59.9% [CI95%: 59.3%–60.5%]. To deliver insights on the set of models before consensus, the proportion of ambiguous clusters (PAC) is reported. This score is computed as the proportion of pairs of patients that were clustered together between 10% and 90% of the time. A lower PAC indicates more stable clusters. The PAC score was 37.9%. This means that 60% of the pair of patients were either always or never together. The detail of the distribution of co-clustered pairs of patients is presented in Supplementary material online, *Figure S1*.

### Merging clusters

**Figure 2:**
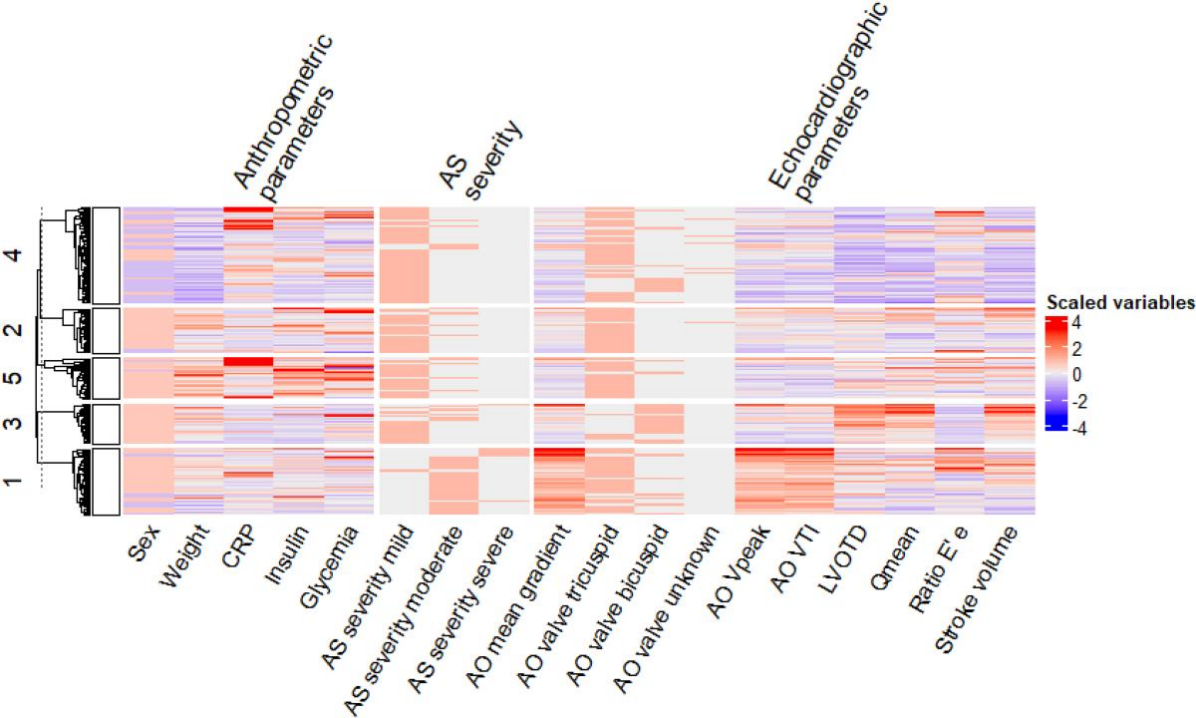
Heatmap of the selected variables and 5 merged clusters of the PROGRESSA study. Rows correspond to each patient’s first visit, grouped according to their final cluster and sorted according to a hierarchical clustering. Columns correspond to the scaled variables by the pipeline. AO, aortic; AS, aortic stenosis; CRP, C-reactive protein; LVOTD, left ventricular outflow tract dimension; VTI, velocity time integral.

Aiming for 10 clusters provides a sufficiently fine-grained perspective on the various phenogroups in the PROGRESSA population. To alleviate the burden of analysis and help clinical interpretation, the fine-grained clusters were merged into five clusters according to their closest centroids as shown in Supplementary material online, *Figure S2*. The final clusters can be seen in *Figure 2* using the top-selected features.

### Comparison of clinical and echocardiographic parameters between clusters

Clinical patient characteristics and laboratory data according to their respective clusters are presented in *Table 1*. AS anatomic and haemodynamic parameters according to clusters are shown in *Table 2*.

**Table 1:** Clinical and laboratory data of patients according to the proposed clusters

	Cluster 1 *N* = 81 (23%)	Cluster 2 *N* = 54 (16%)	Cluster 3 *N* = 47 (13%)	Cluster 4 *N* = 117 (34%)	Cluster 5 *N* = 50 (14%)	*P*-Value
**Clinical Data**						
Age, years	69 [61–75]	72 [63–76]	47 [41–60]	70 [58–75]	69 [63–72]	**<0.001^2,5,8,9^**
Weight, kg *	81 [73–88]	86 [77–94]	82 [77–94]	68 [62–74]	96 [83–107]	**<0.001^1,3,4,6,7,8,9,10^**
BMI, kg/m^2^	28.7 [25.7; 30.5]	30.1 [27.1; 32.1]	27.9 [25.1; 30.5]	26.4 [23.7; 29.2]	32.5 [29.6; 37.0]	**<0.001^3,4,5,6,7,9,10^**
Male sex, *n* (%) *	63 (77)	51 (94)	47 (100)	36 (31)	48 (97)	**<0.001^3,6,8,10^**
Hypertension, *n* (%)	59 (72)	51 (94)	13 (28)	69 (59)	48 (97)	**<0.001^2,5,6,8,9,10^**
Diabetes, *n* (%)	15 (19)	19 (35)	3 (6)	2 (18)	28 (56)	**<0.001^4,5,9,10^**
Coronary artery disease, *n* (%)	23 (28)	29 (54)	2 (4)	25 (21)	23 (46)	**<0.001^1,2,5,6,9,10^**
Bicuspid aortic valve, *n* (%) *	16 (20)	2 (4)	36 (77)	28 (24)	5 (10)	**<0.001^2,5,6,8,9^**
**Laboratory Data**						
Fasting glucose, mmol/L *	5.3 [5.0–6.0]	5.7 [5.2–6.3]	5.2 [4.8–5.8]	5.2 [4.8–5.8]	6.4 [5.5–7.6]	**<0.001^1,4,5,6,7,9,10^**
Insulin, pmol/L *	72 [46–105]	76 [54–116]	51 [38–79]	58 [41–102]	143 [92–177]	**<0.001^2,4,5,6,7,9,10^**
LDL, mmol/L	2.18 [1.90–2.63]	1.91 [1.47–2.49]	2.61 [2.18–3.32]	2.42 [1.86–3.06]	1.75 [1.34–2.07]	**<0.001^1,2,3,4,5,6,7,8,9,10^**
Triglycerides, mmol/L	1.27 [0.98–1.66]	1.49 [0.80–179]	1.01 [0.80–1.33]	1.19 [0.77–1.71]	1.45 [1.17–1.98]	**0.005^9,10^**
Apo B/Apo A ratio	0.55 [0.46–0.65]	0.54 [0.44–0.65]	0.61 [0.48–0.76]	0.53 [0.43–0.66]	0.55 [0.48–0.68]	0.361
Creatinine, µmol/L	84 [74–95]	87 [76–104]	84 [76–90]	72 [56–85]	83 [76–96]	**<0.001^3,6,8,10^**
Nt-proBNP, ng/L	101 [56–252]	132 [42–334]	39 [21–68]	98 [49–183]	85 [40–246]	**<0.001^2,5,8,9^**
CRP, mg/L *	1.65 [0.80–2.60]	0.98 [0.57–2.54]	0.77 [0.42–1.64]	2.27 [0.83–4.71]	2.47 [1.14–8.07]	**<0.001^2,6,7,8,9^**

Apo A, apolipoprotein A-1; Apo B, apolipoprotein B; BMI, body mass index; BSA, body surface area; CRP, C-reactive protein; HDL, high-density lipoprotein; LDL, low-density lipoprotein; Lp(a), lipoprotein(a); Nt-proBNP, N-terminal pro-brain natriuretic peptide. Variables that were selected by more than 90% of the clustering models in the clustering pipeline are presented with *.

**Table:** 

**1** - 1-2 *P* < 0.05 **2** - 1-3 *P* < 0.05 **3** - 1-4 *P* < 0.05	**4** - 1-5 *P* < 0.05 **5** - 2-3 *P* < 0.05 **6** - 2-4 *P* < 0.05	**7** - 2-5 *P* < 0.05 **8** - 3-4 *P* < 0.05 **9** - 3-5 *P* < 0.05	**10** - 4-5 *P* < 0.05

**Table 2:** Echocardiographic and MDCT data and parameters of AS progression according to clusters

	Cluster 1 *N* = 81 (23%)	Cluster 2 *N* = 54 (16%)	Cluster 3 *N* = 47 (13%)	Cluster 4 *N* = 117 (34%)	Cluster 5 *N* = 50 (14%)	*P*-value
**Baseline Echocardiographic and MDCT Data**						
Stroke volume (mL) *	82 [73–90]	80 [72–91]	89 [82–106]	68 [62–75]	77 [73–86]	**<0.001^2,3,5,6,8,9,10^**
Stroke volume index (mL/m^2^)	44 [38–47]	40 [38–44]	45 [42–52]	40 [36–45]	38 [34–42]	**<0.001^1,2,3,4,5,7,8,9,10^**
LA diastolic diameter (mm)	33 [28–37]	34 [30–37]	28 [26–34]	29 [24–32]	34 [31–39]	**<0.001^3,5,6,8,10^**
LV diastolic diameter (mm)	46 [43–49]	47 [45–49]	51 [48–54]	44 [42–46]	49 [47–53]	**<0.001^2,3,4,5,6,8,10^**
Relative wall thickness	0.50 [0.47–0.56]	0.49 [0.44–0.54]	0.44 [0.39–0.49]	0.48 [0.43–0.54]	0.45 [0.40–0.52]	**<0.001^2,4,5^**
V_peak_ (cm/s) *	344 [314–376]	262 [247–289]	255 [234–287]	251 [231–276]	249 [235–264]	**<0.001^1,2,3,4^**
MG (mmHg) *	28.2 [23.3–33.1]	15.1 [13.3–18.2]	15.2 [11.8–20.0]	14.2 [11.6–16.4]	13.6 [12.0–16.9]	**<0.001^1,2,3,4^**
AVA (cm^2^) *	0.97 [0.85–1.08]	1.31 [1.11–1.47]	1.48 [1.34–1.70]	1.17 [1.04–1.34]	1.43 [1.22–1.60]	**<0.001^1,2,3,4,5,8,10^**
AVAi (cm^2^/m^2^)	0.50 [0.46–0.58]	0.65 [0.58–0.74]	0.75 [0.67–0.86]	0.67 [0.61–0.78]	0.68 [0.56–0.77]	**<0.001^1,2,3,4,5,9^**
AS severity * (mild)	3 (3)	46 (85)	34 (72)	106 (91)	44 (88)	**<0.001^1,2,3,4,8,9^**
(moderate)	67 (83)	8 (15)	12 (26)	11 (9)	6 (12)	
(severe)	11 (14)	0 (0)	1 (2)	0 (0)	0 (0)	
LVOT diameter (mm) *	22.4 [21.3–23.4]	22.4 [21.5–23.3]	25.0 [23.1–27.0]	20.4 [19.4–21.3]	22.4 [21.5–23.8]	**<0.001^2,3,5,6,8,9,10^**
Qmean (ml/s) *	239 [218–265]	232 [213–278]	295 [256–348]	208 [187–235]	250 [227–270]	**<0.001^2,3,5,6,8,9,10^**
E/e′ ratio *	11.3 [9.2–15.5]	10.5 [9.1–13.3]	8.1 [7.6–9.3]	10.9 [8.4–13.6]	11.3 [9.1–14.8]	**<0.001^2,5,8,9^**
LVEF (%)	65 ± 5	63 ± 6	65 ± 5	67 ± 5	62 ± 6	**<0.001^6,8,10^**
AVC score (UA)	1257 [806–1837]	739 [537–1053]	708 [281–1480]	374 [163–588]	358 [230–731]	**<0.001^3,4,6,8^**
**AS Annualized Progression Parameters**						
Delta V_peak_	22 [9–39]	11 [3–19]	11 [3–22]	7 [3–17]	8 [0–14]	**<0.001^1,3,4^**
Delta MG	4.4 [2.0–7.7]	1.4 [0.4–3.2]	1.3 [0.1–2.9]	1.1 [0.4–2.7]	1.0 [0.3–2.3]	**<0.001^1,2,3,4^**
Delta AVA	−0.08 [−0.13 to −0.04]	−0.05 [−0.08 to −0.01]	−0.05 [−0.09 to −0.01]	−0.04 [−0.08 to −0.01]	−0.05 [−0.09 to 0.01]	**0.002^1,2,3^**
Delta AVC score	213 [111–307]	93 [35–140]	102 [24–311]	64 [11–121]	61 [24–156]	**0.001^1,3,4^**

AVA, aortic valve area; AVAi, indexed aortic valve area; AVC score, aortic valve calcium score; LA, left atrium; LV, left ventricle; LVEF, left ventricular ejection fraction; LVOT, left ventricular outflow tract; MG, mean gradient; V_peak_, peak aortic jet velocity. Variables that were selected by more than 90% of the clustering models in the clustering pipeline are presented with *.

**Table:** 

**1** - 1-2 *P* < 0.05 **2** - 1-3 *P* < 0.05 **3** - 1-4 *P* < 0.05	**4** - 1-5 *P* < 0.05 **5** - 2-3 *P* < 0.05 **6** - 2-4 *P* < 0.05	**7** - 2-5 *P* < 0.05 **8** - 3-4 *P* < 0.05 **9** - 3-5 *P* < 0.05	**10** - 4-5 *P* < 0.05

Patients in cluster 1 had higher initial AS haemodynamic severity^29^ (V_peak_ = 344 [4–376] cm/sec, MG = 28.2 [23.3–33.1] mmHg and AVA = 0.97 [0.85–1.08] cm^2^, all *P* < 0.001) and calcific burden (AVC score = 1257 [806–1837] UA, *P* < 0.001) at baseline than the other clusters. Patients in cluster 2 were majority male (*n* = 51, 94%) but had no other discernible characteristics. Patients in cluster 3 were younger (age = 47 [41–60] years, *P* < 0.001), exclusively male and had a higher proportion of bicuspid valves (*n* = 36, 77%). Cluster 4 consisted mainly of female patients (*n* = 81, 69%). Patients in cluster 5 had a higher BSA (2.10 [1.97–2.20] m^2^, *P* < 0.001) and more cardiovascular co-morbidities such as hypertension (*n* = 48, 97%), diabetes (*n* = 28, 56%), and coronary artery disease (*n* = 23, 46%). They also had an overall worse cardiometabolic profile with higher fasting glucose (6.4 [5.5–7.6] mmol/L, *P* < 0.001), insulin (143 [92–177] pmol/L, *P* < 0.001), and CRP (2.47 [1.14–8.07] mg/L, *P* < 0.001).

### AS progression in the clusters

**Figure 3:**
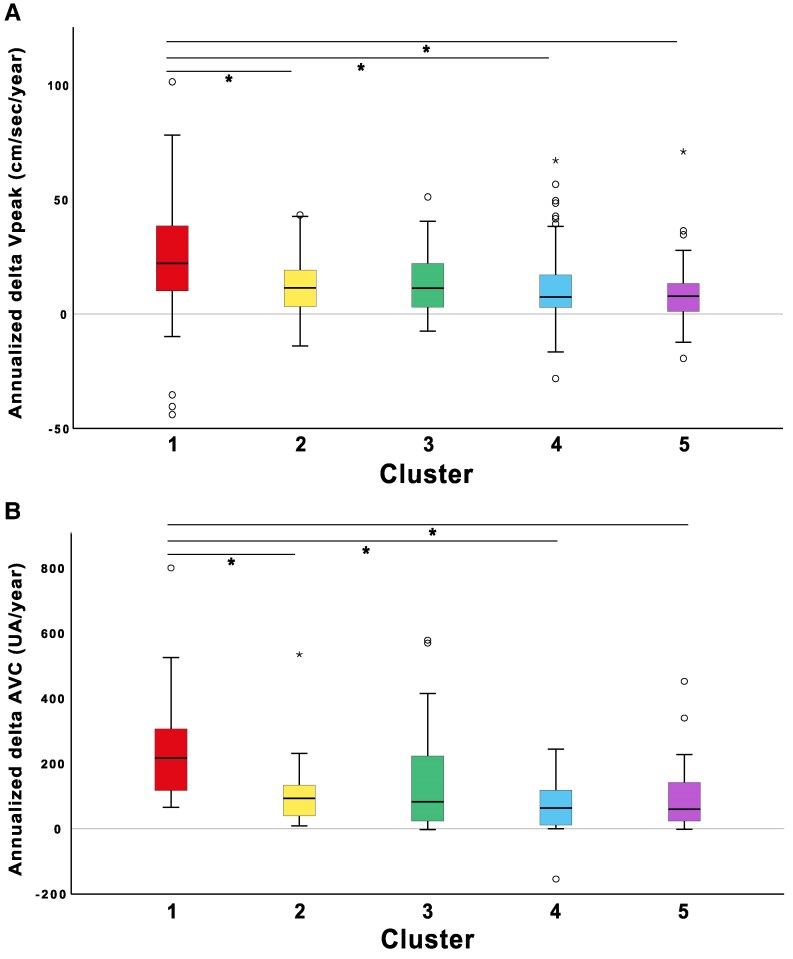
Haemodynamic and anatomic progression of AS according to clusters. Box-plot representation of (*A*) Annualized Vpeak progression and (*B*) Annualized AVC progression of AS according to different clusters. In panel A, Vpeak progression is significantly different between clusters 1–2, 1–4 and 1–5. In panel B, AVC progression is significantly different between clusters 1–2, 1–4 and 1–5. With cluster 1 having a faster progression in all cases. AVC, Aortic Valve Calcification; Vpeak, Peak Aortic Valve Velocity. * = *P* < 0.05.

Follow-up echocardiographic analysis was available in 305 (87%) of patients with a median follow-up time of 3.89 [2.00–5.13] years. Annualized AS progression rates were similar between clusters except for cluster 1 (*Table 2*). Patients in cluster 1 had significantly faster AS progression rate according to delta V_peak_ (22 [9; 39], *P* < 0.001) cm/sec, delta MG (4.4 [2.0; 7.7], *P* < 0.001) mmHg, delta AVA (0.08 [−0.13; −0.04], *P* < 0.001) cm^2^, and delta AVC score (213 [111; 307], *P* = 0.001) AU. The differences in the progression rate of AS between each cluster can be observed in *Figure 3*.

### Clinical outcomes in the clusters

**Figure 4:**
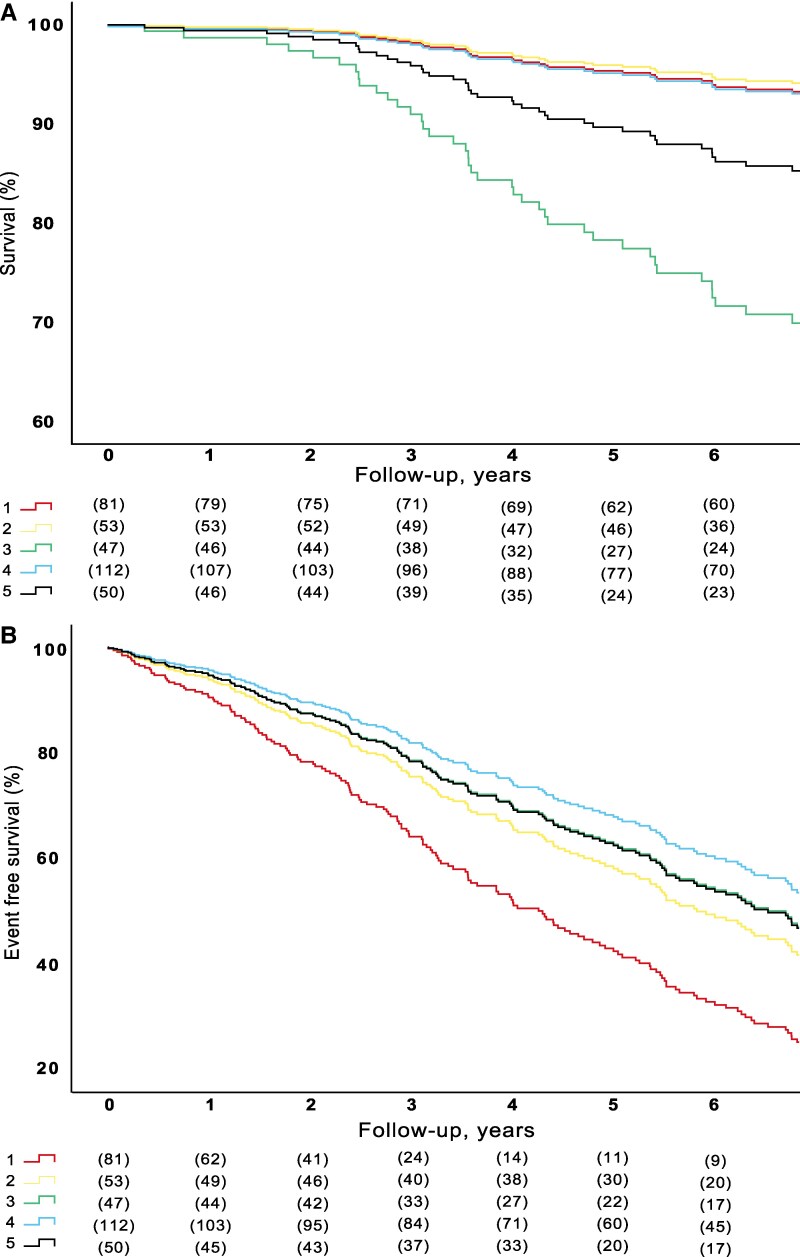
Adverse outcomes according to the different clusters. Cox regression curves adjusted for age, sex and Vpeak. (*A*) Shows the association between clusters and all-cause mortality and (*B*) the composite endpoint of all-cause mortality and AVR.

During a median follow-up time of 7.4 [4.3–9.3] years, 66 (19%) patients died, and 188 (54%) patients met the composite endpoint of death or AVR. Unadjusted curves for mortality and the composite endpoint of AVR and mortality are presented in Supplementary material online, *Figure S3*. Cox proportional hazards regression curves (adjusted for age, sex and initial V_peak_ as an indicator of AS severity) were performed for both all-cause mortality and a composite endpoint of AVR and all-cause mortality (*Figure 4*). There was a significant difference between clusters regarding mortality, with patients from cluster 3 having increased mortality (*P* < 0.001) when compared with other clusters. Subsequently, the analysis regarding the composite endpoint showed that patients from cluster 1 had an increased risk of the composite endpoint when compared with the other clusters (*P* < 0.001). Additionally, patients from cluster 5 had a slightly lower increase of the composite endpoint but were still significantly different from the other clusters (*P* < 0.001).

### Performance of the logistic regression for deployment

**Figure 5:**
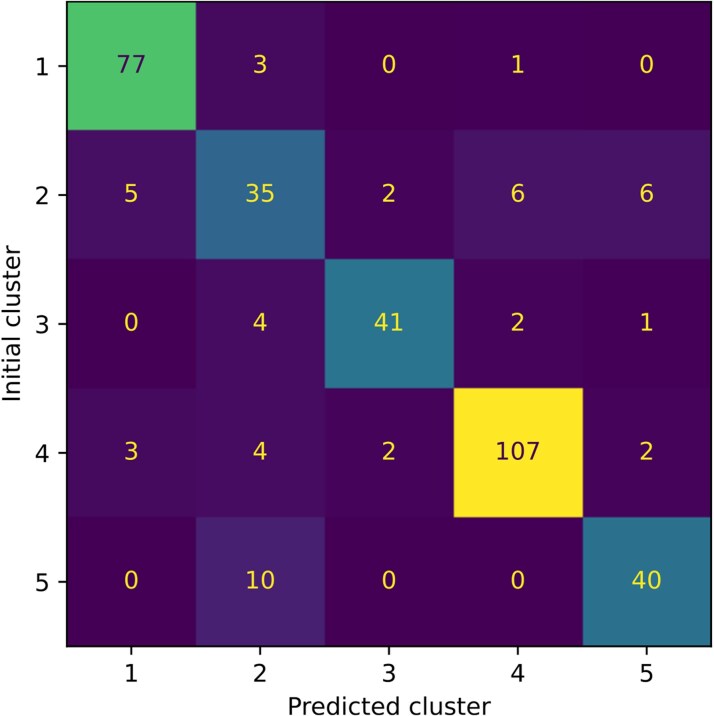
Confusion matrix of the logistic regression trained with the top-selected variables including the CRP on the set of the first visits of patients. The rows correspond to the clusters that were discovered by the unsupervised pipeline. The predicted clusters on the columns correspond to the class that was predicted by the logistic regression. The reached accuracy was 85.5%.

**Figure 6:**
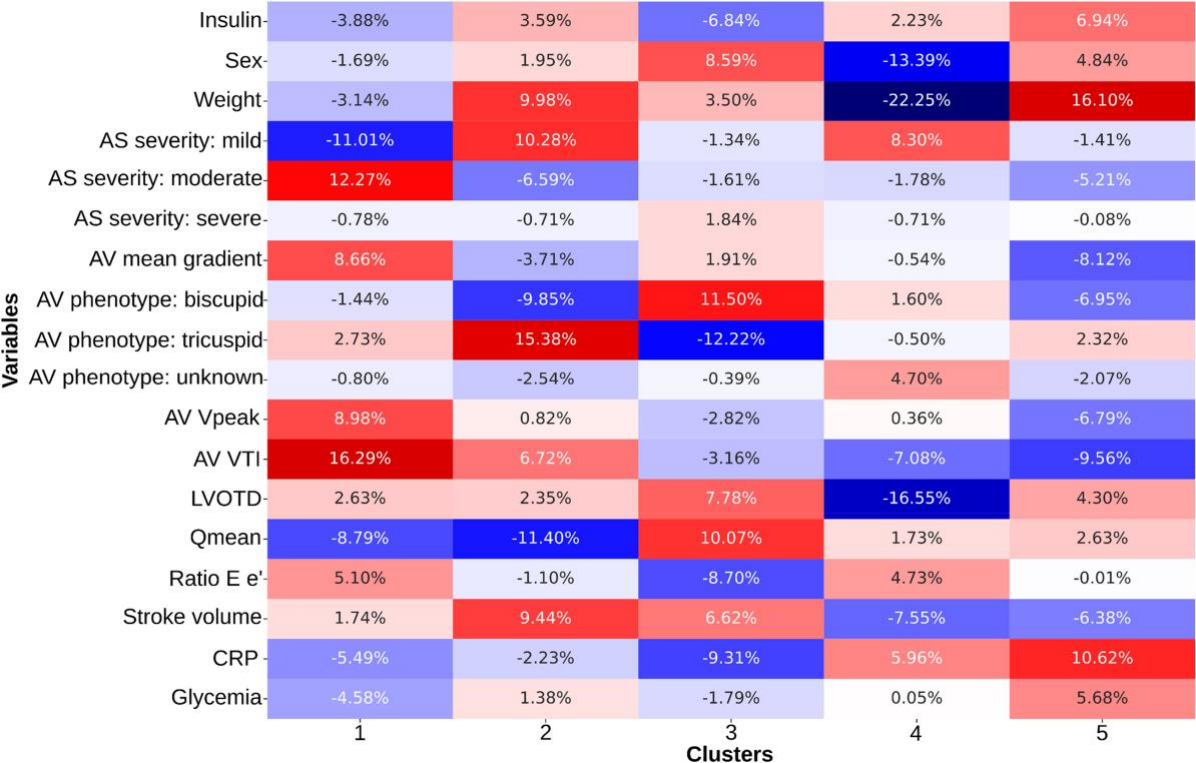
Normalized weights of the logistic regression with the top-selected variables including CRP on the set of the first visits of patients. Absolute weights add up to 100% per cluster. Positive values highlight a positive correlation between the final class and negative values a negative correlation. AS, aortic stenosis; AV, aortic valve; CRP, C-reactive protein; LVOTD, left ventricular outflow dimension tract; VTI, velocity time integral.

The logistic regression, i.e. the end model that aims at predicting the cluster to which novel patients will belong, was trained using the subset of variables indicated in *Figure 2*. *Figure 5* shows the confusion matrices of the final models after the grid search on the complete dataset using only the first visits. The end model reached a cross-validation f1 score of 0.811 (95 CI: 0.799–0.822) for an accuracy of 85.5% on the complete dataset. In case of a missing CRP, we treat it as a missing variable and impute it with the mean of the CRP distribution. When CRP is replaced by the mean value, the performances of the model are up to 0.821 of f1-score and an accuracy of 82.6% on the complete dataset. Finally, we report in *Figure 6* the normalized weights of the logistic regressions per class. These weights account for the most characteristic variables of each recovered class when they have a high positive or negative value. The absolute weights add up to 100%.

Cluster 1 is strongly influenced by the presence of moderate AS, and thus the aortic valve VTI and Vpeak, but is not affected by the mean flow rate. Similarly, cluster 2 is characterized by mild AS, tricuspid valve phenotype, and patient weight. This cluster uniquely emphasizes the tricuspid valve, which may account for misclassifications observed in *Figure 5* due to the overemphasis on this variable. Cluster 3 primarily relies on the sex variable and Qmean in patients with a bicuspid valve, with minimal influence from CRP. Cluster 4 focuses on mild AS and excludes contributions from sex, weight, and left ventricular outflow tract diameter. Lastly, cluster 5 is predominantly associated with CRP and weight, while reducing the influence of aortic valve velocity time integral and MG.

## Discussion

The main findings of this study were that: (ⅰ) By using unsupervised cluster analysis, 5 clusters with significantly different clinical characteristics were obtained in a cohort of patients with mild to severe AS, (ⅱ) The annualized progression rate was different between clusters, and (ⅲ) Survival and intervention assessment of the clusters show increased mortality rates and AVR according to the phenogroup classification.

### Phenogroup identification for AS

Risk stratification while determining the optimal timing and course of treatment for AS presents a significant challenge for clinicians due to the condition's heterogenic presentation. The recommended treatment, surgical or transcatheter AVR, is an invasive procedure that carries inherent risks. In this retrospective study of 349 AS patients, we developed a model integrating clinical and blood data and standard non-invasive echocardiographic parameters that attempts to characterize different AS groups that could be at higher risk of mortality or AVR.

Few studies used unsupervised machine learning in the field of valvular heart diseases*. Kwak et al.*^18^ proposed the use of clustering for the development and validation of a new classification of AS, but they included patients with moderate and severe AS neglecting patients with mild AS. They found three clusters that differed in cardiac function and co-morbidities. A recent study by *Lachmann et al.*^20^ used a phenomapping technique to produce four AS mutually exclusive classification phenogroups with an increased mortality according to the phenogroup. They also found that the prognosis of severe AS was more impacted by the extra-valvular cardiac damages than the degree of AS. Despite being a robust and large study, the focus was solely made on severe AS, consequently not representing the whole spectrum of the disease. In our study, we studied a real-life population of patients with mild to severe AS with a long-term follow-up of the progression of the disease. Finally, we used all-cause mortality as a strong primary endpoint, as well as a composite endpoint of all-cause mortality and AVR.

Other works focused on identifying phenogroups for AS using clustering methods. These methods often tried to seek phenotypes qualifying the severity of AS^16^ or qualifying the survival rates of different populations.^18^ These methods often access a population of patients of size comparable to PROGRESSA with a few hundred samples.

Indeed, other AS phenotyping works rather start with variable selection using PCA as a variable importance criterion and co-linear variables removal according to Pearson correlation.^18,20^ The PROGRESSA study collects comprehensive clinical, imaging, and metabolic data which, unlike prior studies, allows for the inclusion of a much larger number of features and provides the advantage of identifying new features of AS progression and outcomes not previously identified. The number of initial variables before selection is lower than in our study: from 5^17^ to 60.^19^ The clustering algorithms then range from ward agglomerative clustering^20^ to model-based clustering (using *mclust* in R)^18^ passing through topological analysis.^19^

The previously published clusters often highlight phenotypes with severe AS and low survival rates.^17,18^ Those phenotypes present extensive disease characteristics such as high NYHA score and impaired cardiac functions,^20^ and potentially severe AS with one or several co-morbidities, especially among the older population.^18,19^ Finally, other groups were characterized by a younger population still through healthy AS. Our study brings further evidence of AS co-morbidities and the central role of the valve bicuspid profile in survival.

It is important to note that the consensus clustering algorithm at the end of the pipeline is non-parametric. This implies that the pipeline cannot generalize to unseen samples, thus justifying the introduction of the supervised models for reusability purposes. There is therefore a completeness between the contributions on the identification/corroboration of novel knowledge through clustering and on the future predictions for unseen patients.

### Clinical interpretation of the clustering model findings

The pathology and clinical manifestations of AS are multifaceted. In this study, machine-learning analysis successfully identified significant risk subgroups within AS, validating previous findings that suggested the potential of machine learning in unravelling the diverse phenotypic presentations of AS. We were able to identify distinct phenogroups of patients with mild to severe AS who had specific characteristics. Cluster 1 had a higher initial degree of AS haemodynamic and anatomic severity; Cluster 2 was mainly constituted by males with no other discernible characteristics; Cluster 3 was predominantly composed of young males with a high proportion of bicuspid aortic valves; Cluster 4 was composed of a higher percentage of female patients; and Cluster 5 was characterized by patients with a higher percentage of obesity and cardiovascular co-morbidities. Cluster 1 exhibited a faster AS progression rate and higher risk of the composite of AVR or mortality. Cluster 3 presented the highest risk of mortality after adjusting for age and sex.

The increased mortality and AVR in patients from Cluster 1 could be explained by the fact that they had a significantly more severe degree of AS initially. It is well known that one of the most important factors associated with the progression of AS is a higher baseline severity of AS.^30^ Also, if AS is more severe for some patients, they will reach haemodynamic thresholds for surgery faster than patients with less severe AS.

Excluding Cluster 1, all clusters had similar progression rates of AS. The prediction of AS progression remains a challenge and, in this case, machine learning could not provide insight into faster progression.

Sex differences in the development, progression, and clinical presentation of valvular diseases have been well-documented in past studies.^31,32^ Our results support these observations with the significant distribution of females in a single cluster: cluster 4. This reinforces the notion that female patients with valvular disease should have personalized clinical management and eventually a treatment specific to the sex-specific physiopathology of AS in women.

The reduced survival in Cluster 3, predominantly composed of men with BAV emphasizes the importance of careful follow-up and optimized management in this subset of patients. Interestingly, this group did not present with more severe AS, a worse comorbidity burden nor with clearly adverse cardiac remodeling compared with others. This raises the hypothesis that traditional parameters may not fully capture risk in bicuspid AS. While the underlying mechanisms remain uncertain, these findings suggest that bicuspid AS may follow a distinct disease trajectory, warranting closer clinical attention and further investigation. In this group there were four deaths. The causes of death in this group (cardiac decompensation, lung cancer, and undetermined) were heterogeneous and do not point to a single mechanism. These findings highlight the complexity of risk assessment in BAV and the potential value of integrative, phenotype-based approaches to better identify vulnerable patients.

This machine learning model was able to identify distinct phenotypes of AS that were clinically sound, highlighting that using this technique could be used to differentiate groups of patients with this disease. An approach like this could potentially be used when developing pharmacological therapies for AS, targeting the pathological profile of specific patients. This model offers a structured approach to explore and document patterns of comorbidity and clinical characteristics in patients with AS. This contributes to a better understanding of the diversity and complexity of multimorbidity in real-world scenarios. In the future, other imaging modalities as well as proteomic data could be added to machine learning models to obtain a more refined phenogrouping of patients and even better survival prediction.

### Study limitations

Despite the prospective collection of clinical, laboratory, and echocardiographic data, the analysis was performed retrospectively, which introduces certain limitations inherent to this approach. One important limitation is the high proportion of missing data across the initial dataset: over 82% of the variables had more than 5% missing values and were therefore excluded from the analysis. This step, while necessary to ensure data quality and prevent biased clustering, reduced the number of variables available for the final model. Among the retained variables (i.e. those with < 5% missing data), we performed a complete case analysis. While this approach is widely accepted when missingness is low, it can still introduce bias if the excluded cases are systematically different from the rest of the population.

Additionally, the cohort used in this study could be considered small for working with machine learning. Indeed, larger cohorts are usually used in this setting, but that fact is offset by the quality and extent of patient characterization. The data collected in this study gives us a complete overview of the patient’s clinical and metabolic determinants as well as long-term disease progression evaluation which is rather rare in this type of study. To validate these findings, an independent external cohort could be used to confirm the reproducibility of our findings.

The prospective nature of the PROGRESSA study can also create limitations regarding follow-up data. Some patients did not have all metabolic, echocardiographic or MDCT data for all possible follow-ups.

The proportion of women included in this analysis is also small, once again limiting the ability to generalize our findings to this specific group. The fact that most women are classified in the same cluster is however encouraging and would suggest that if more women were included, they would belong to the same cluster and merit specific risk stratification and therapeutical management.

The primary endpoints chosen could also be criticized considering that the occurrence of AVR is largely determined by the clinician’s perception of disease severity and could be a confounding factor for mortality. Patients who underwent AVR can see their life expectancy increased, thus affecting the mortality endpoint. To counteract this bias, we decided to present two separate endpoints, mortality and a composite endpoint of mortality and AVR, to better represent the prognosis and treatment of patients with AS. Given data availability, all-cause mortality was used as a primary endpoint in this study. However, we recognize that its low frequency in this cohort and the potential for confounding causes of death may limit interpretability. Furthermore, our model incorporated only a limited set of non-echocardiographic variables. This raises the possibility that additional unmeasured or unknown factors may have contributed to residual confounding and could also be responsible for the low characterization of the patients in Cluster 3.

We did not have a validation cohort to confirm our findings, thus limiting our ability to generalize our conclusions. We however developed the supervised algorithm and cross-validation on the dataset to ensure that the clusters could be found and generalized using fewer samples in the training set. The weights of the logistic regression are available upon request.

## Conclusion

This study demonstrated meaningful differences in AS severity, clinical data and prognosis across five distinct clusters identified by a machine learning algorithm. Patients in cluster 1 had a higher initial degree of AS severity, presented a faster AS progression rate and higher risk of the occurrence of AVR during follow-up. Patients in cluster 2 were mainly males but had no other distinctive characteristics. Patients in cluster 3 were predominantly younger, males with bicuspid aortic valve and these patients were at higher risk of mortality despite a more favorable risk profile at baseline compared with other clusters. Cluster 4 was mainly composed of female patients. Patients in cluster 5 had a higher prevalence of obesity and cardiometabolic risk factors. These findings emphasize the importance of close clinical surveillance and potentially of earlier valve intervention, in the subset of male bicuspid patients. In the absence of effective tools to better stratify the risk and therapies to improve prognosis in patients with AS, the separation of individuals into clinically distinct phenogroups could help identify patients who could benefit from targeted interventions and therapies. Due to risk factors and outcomes in patients with AS, future trials should concentrate on various interventions tailored to specific phenogroups within this patient population.

## Supplementary Material

ztaf115_Supplementary_Data

## Data Availability

The data underlying this article will be shared on reasonable request to the corresponding author.
