# Supplementary material for: Unsupervised machine learning analysis to enhance risk stratification in patients with asymptomatic aortic stenosis
Source: Eur Heart J Digit Health. 2025 Oct 9;7(1):ztaf115. doi: 10.1093/ehjdh/ztaf115 (PMC12821062; doi:10.1093/ehjdh/ztaf115)
Supplement: ztaf115_Supplementary_Data [file ztaf115_supplementary_data.pdf]

Online supplement

Unsupervised Machine Learning Analysis to Enhance Risk Stratification in Patients with Aortic Stenosis

Marie-Ange Fleury‡, MSc; Louis Ohl‡, PhD; Lionel Tastet, PhD; Mickaël Leclercq, PhD; Frédéric Precioso, PhD, Pierre-Alexandre Mattei[5], PhD; Romain Capoulade, PhD; Kathia Abdoun, Msc; Élisabeth Bédard, MD; Marie Arsenault, MD; Jonathan Beaudoin, MD; Mathieu Bernier, MD; Erwan Salaun, MD, PhD; Jérémy Bernard, PhD; Mylène Shen, PhD; Sébastien Hecht, PhD; Nancy Côté, PhD; Arnaud Droit*, PhD; Philippe Pibarot*, DVM, PhD

‡ MAF and LO are joint first author.

* PP and AD are joint corresponding authors.

Considered Datasets and Preprocessing

The PROGRESSA study database contains clinical, metabolic, biological, echocardiographic, and MDCT data. As the goal is to find stable clusters of phenogroups per patient, i.e. patient clusters that are invariant to the number of visits, the dataset was divided into two subsets: the subset of the first patients' visits and the subset of the subsequent visits. Indeed, as we expected clusters to be invariant over time, the first visit of each patient was sufficient to determine the phenogroup. Variables that have more than 5% of missing values were dropped. The missing values of the remaining variables are replaced either by the mean or the most frequent term depending on the type of variable. Categorical variables were transformed with one-hot-encoding and continuous variables with robust scaling. The specific interest of robust scaling is its preservation of outliers: this avoids compression of data in a specific region of space as the standard scaling would do. This scaling of variables was fitted only on the variables of the first visits, and then applied to other visits.

Base Clustering

To perform clustering, we sought to use a discriminative method (i.e. no parametric hypothesis on the data distribution and direct identification of clusters) and incorporate a feature selection mechanism inside the model to keep a few variables that are relevant for interpreting the clusters. We identified the generalized mutual information (1) (GEMINI) for clustering: an information-theoretic score for discriminative clustering. GEMINI uses distances between data samples to guide the clustering and leverages training of neural networks (flexible for decision boundaries in the date) and non-parametric models. The models trained with GEMINI can generalize to unseen samples helping in filtering results based on the clustering of subsequent visits. A sparsity-constrained GEMINI (2) was used to identify 10 clusters using either a linear regression or a multi-layered perceptron (3), i.e. a neural network. We used both the one-vs-all and one-vs-one maximum mean discrepancy (MMD, a measure used to compare two data distributions using means) GEMINI. This number of clusters is sufficiently high to deliver a

fine-grained partition of the patients, e.g. distinguishing patients that belong to a single phenogroup from those that belong to two phenogroups at the same time. Each combination of loss and architecture was run 30 times (i.e. trained on the first visits) resulting in 120 different models. Each model provided a clustering of the patients' first visit and a subset of selected features to obtain these clusters. The selected subsets of features allow one to count the number of times a feature was selected, 120 times at most. Features that were successfully selected more than 108 times (90% of the runs) were kept and used for the heatmap visualization of the clusters. Sparse GEMINI is provided by the GemClus Python package (4).

Results Filtering

To identify stable-over-time clusters, each model was used to cluster the remaining visits of every patient. Then, the first visit cluster was used as a ground truth label, allowing us to measure the accuracy of the remaining visits' clusters. Both the visit-wise accuracy and the patient-wise accuracy that accounts for the imbalance of visits between all patients are reported. Visit-wise accuracy is the average number of visits that were successfully in the same cluster as their respective first visit. In patient-wise accuracy, each group of visits of a patient is divided by the number of visits in this group. Visit-wise accuracy is always greater than patient-wise accuracy. Models that achieve a patient-wise accuracy greater than 90% of the best accuracy of clusters were kept.

Consensus Clustering

To conclude on a final clustering, consensus clustering was used (5). The consensus matrix was built by computing the ratio of times two samples were clustered together among the most accurate models (6). The nonparametric Wasserstein-OvO GEMINI model (1) was used for the final clustering. The benefit of this model is that the parameters can draw any kind of decision boundary without requiring the value of the samples: only the connectivity between samples is considered. The complete picture of the clustering process is summarized in **Figure 1**.

Building a Supervised Model for Deployment

The nonparametric consensus model cannot generalize to unseen samples. Consequently, the pipeline cannot be applied to new patients. To provide a model ensuring the reproducibility of results, a ridge regression was trained to recover the identified clusters. Since the pipeline ends with a nonparametric consensus clustering algorithm, the optimal decision boundaries may be nonlinear, in which case the logistic regression may not reach 100% accuracy. However, the strong advantage of this algorithm is the interpretability of its weights. The implementation uses the logistic regression provided by the scikit learn Python package (7).The parameters of the model were selected based on a grid search. The regularization strength varied between 10^{-3} and 10^{2}, and the tolerance threshold for the convergence varied between 0 and 0.1. Models were evaluated with the F1 score. Cross-validation was performed over 50 randomly stratified shuffled splits.

References

1. Ohl L, Mattei P-A, Bouveyron C, Harchaoui W, Leclercq M, Droit A, et al. Generalised Mutual Information for Discriminative Clustering. Advances in Neural Information Processing Systems. 2022;35.

2. Ohl L, Mattei P-A, Bouveyron C, Leclercq M, Droit A, Precioso F. Sparse and geometry-aware generalisation of the mutual information for joint discriminative clustering and feature selection. Statistics and Computing. 2024;34(5):26.

3. Lemhadri I, Ruan F, Abraham L, Tibshirani R. LassoNet: A Neural Network with Feature Sparsity. Journal of Machine Learning Research. 2021;22(127):1-29.

4. L. O, PA. M, F. P. GemClus Published online November 20232023 [

5. Strehl A, Ghosh J. Cluster Ensembles --- A Knowledge Reuse Framework for Combining Multiple Partitions. J Mach Learn Res. 2002;3:583-617.

6. Monti S, Tamayo P, Mesirov J, Golub T. Consensus Clustering: A Resampling-Based Method for Class Discovery and Visualization of Gene Expression Microarray Data. Machine Learning. 2003;52(1):91-118.

7. Pedregosa F, Varoquaux G, Gramfort A, Michel V, Thirion B, Grisel O, et al. Scikit-learn: Machine Learning in Python. Journal of Machine Learning Research. 2012;12.

Online Table 1 - Whole Cohort Clinical and Metabolic Data

	**Whole Cohort n=349**
**Clinical Data**	
Age, years	68 [57-74]
Weight, kg *	79 [70-89]
BMI, kg/m^2	28.4 [25.6; 30.9]
Male sex, n (%) *	245 (70)
Hypertension, n (%)	240 (69)
Diabetes, n (%)	86 (25)
Coronary artery disease, n (%)	102 (29)
Bicuspid aortic valve, n (%) *	87 (25)
**Metabolic Data**	
Fasting glucose, mmol/L *	5.4 [5.0-6.1]
Insulin, pmol/L *	71 [46-113]
LDL, mmol/L	2.18 [1.75-2.78]
Triglycerides, mmol/L	1.27 [0.90-1.73]
Apo B/Apo A ratio	0.54 [0.46-0.66]
Creatinine, µmol/L	80 [70-94]
Nt-proBNP, ng/L	86 [42-210]
CRP, mg/L *	1.53 [0.70-3.48]

Online Table 2 - Whole Cohort Echocardiographic and MDCT data

	**Whole Cohort n=349**
**Baseline Echocardiographic and MDCT Data**	
Stroke volume (mL) *	80 [68-86]
Stroke volume index (mL/m^2)	41 [37-46]
V_{peak} (cm/s) *	269 [242-309]
MG (mmHg) *	15.8 [12.9-22.5]
AVA (cm^2) *	1.21 [1.02-1.44]
AVAi (cm^2/m^2)	0.65 [0.55-0.75]
AS severity * (mild)	233 (67)
(moderate)	104 (30)
(severe)	12 (3)
LVOT diameter (mm) *	22.0 [20.7-23.3]
Qmean (ml/s) *	237 [207-267]
E/e' ratio *	10.3 [8.5-13.1]
LVEF (%)	65±6
AVC score (UA)	600 [295-1075]
**Aortic Stenosis Annualized Progression Parameters**	
Delta V_{peak}	11 [3-22]
Delta MG	1.5 [0.4-3.7]
Delta AVA	-0.05 [-0.10- -0.01]
Delta AVC score	84 [25-159]

V_{peak}; peak aortic jet velocity, MG; mean gradient, AVA; aortic valve area, AVAi; indexed aortic valve area, LV; left ventricle, LVEF; left ventricular ejection fraction, AVC score; aortic valve calcium score. Variables that were selected by more than 90% of the clustering models in the clustering pipeline are presented with *.

Online Figure 1 - Cumulative Distribution Function (CDF) of the agreement frequency between pairs of patients for the 33 Most Accurate Models

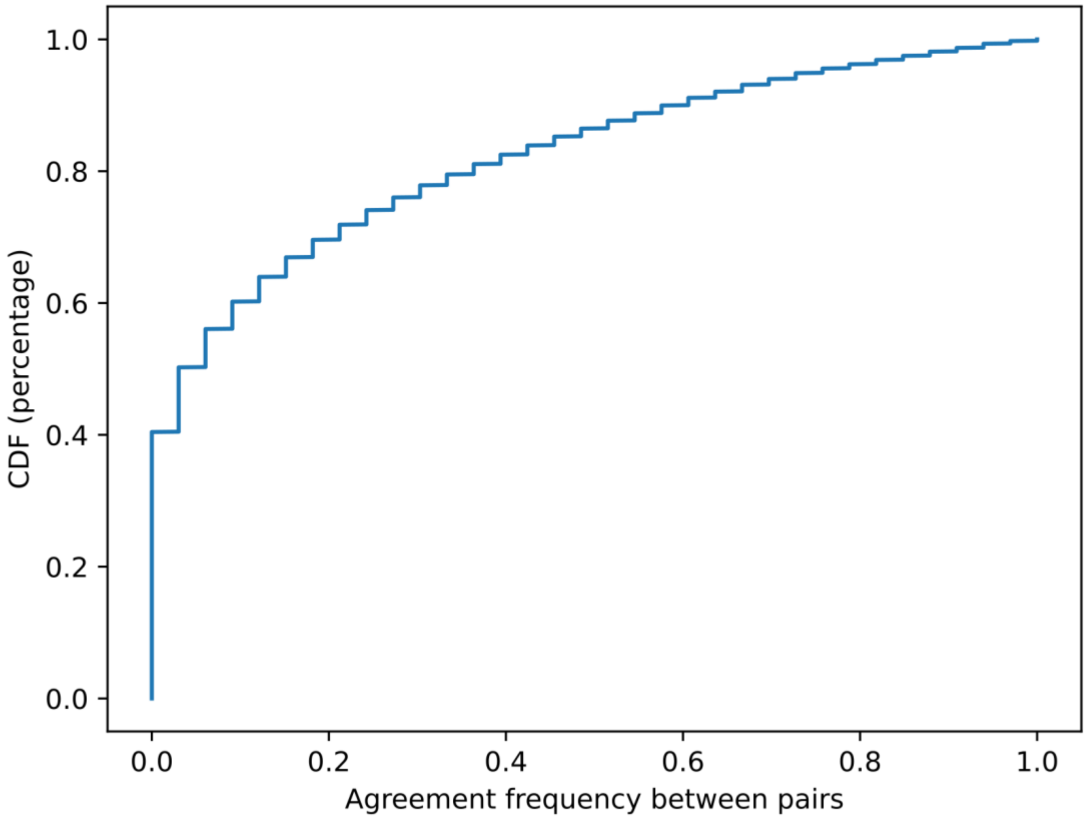

Caption: Forty (40)% of the pairs of patients are never clustered together. One to two (1-2)% of the pairs of patients are always clustered together. Thirty (30)% of the pairs of patients are clustered together with a probability between 20% and 80%.

Online Figure 2 - Distances Between Centroids of all 10 Clusters (0 to 9) in the Final Clustering

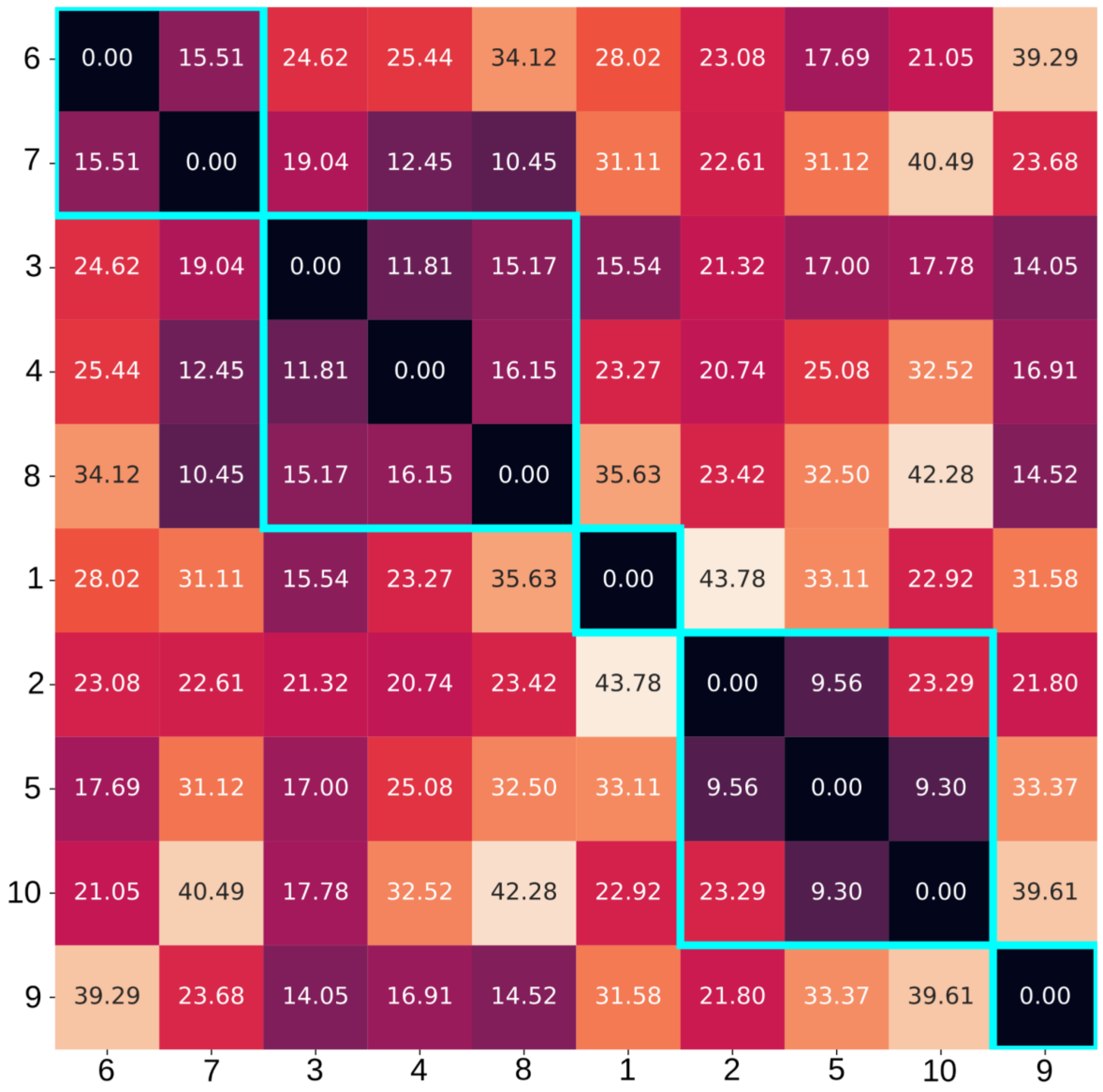

Caption: The ticks indicate the original cluster label and the new 5 merged clusters correspond to the cyan squares. For instance, clusters 5 and 6 were merged.

Online Figure 3 – Kaplan Meier Curves for the Composite Endpoint of Mortality and Aortic Valve Replacement According to Clusters

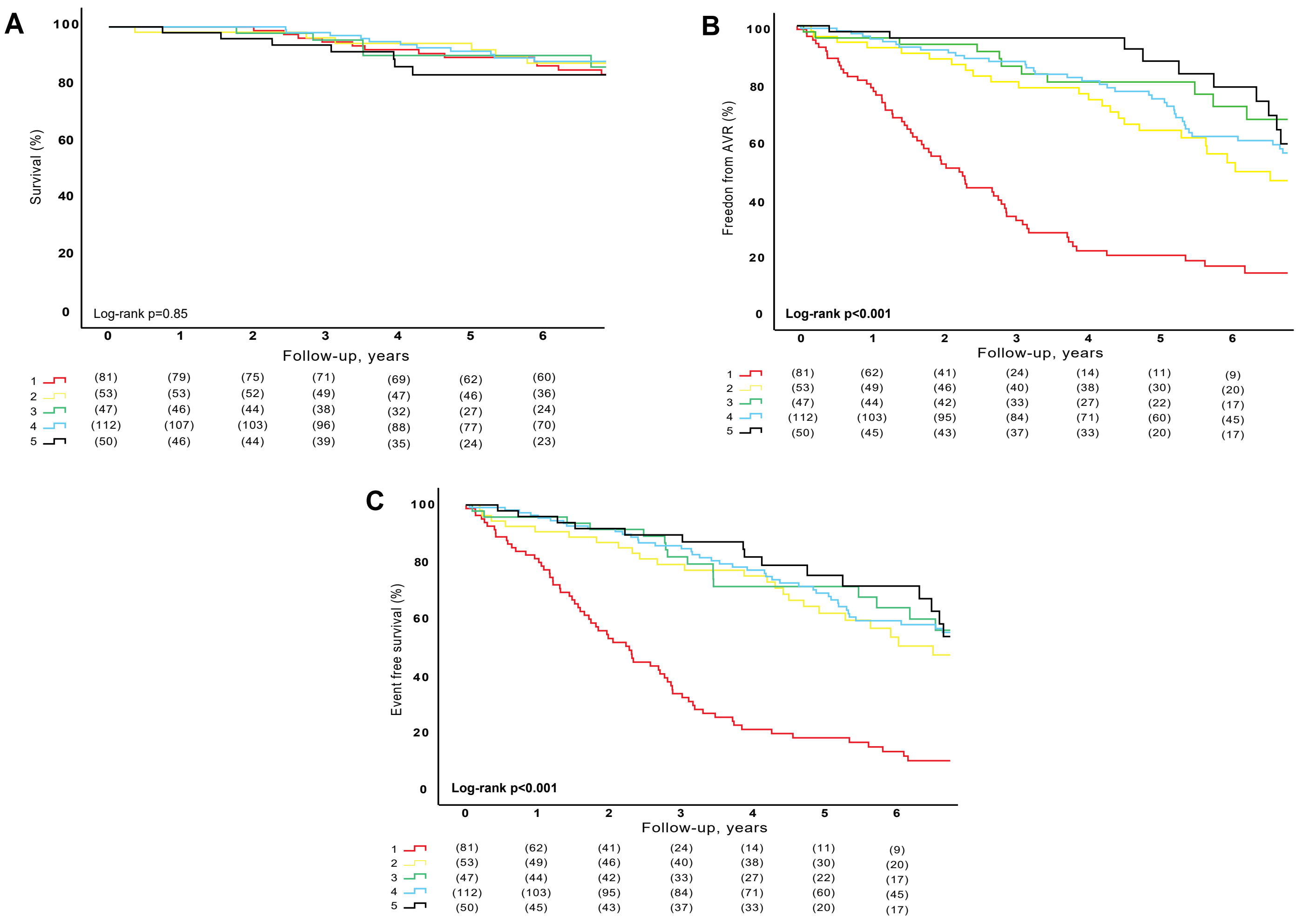

Caption: Kaplan Meier curves showing the association between clusters for (A) all-cause mortality, (B) aortic valve replacement and (C) the composite endpoint of all-cause mortality and aortic valve replacement
